# Extracellular Vesicles, as Drug-Delivery Vehicles, Improve the Biological Activities of Astaxanthin

**DOI:** 10.3390/antiox12020473

**Published:** 2023-02-13

**Authors:** Young Jun Jang, Byung Seok Cha, Doyeon Kim, Eun Sung Lee, Seokjoon Kim, Jinjoo Han, Jiye Shin, Seokhwan Kim, Ki Soo Park

**Affiliations:** Department of Biological Engineering, College of Engineering, Konkuk University, Seoul 05029, Republic of Korea

**Keywords:** astaxanthin (AST), extracellular vesicles (EVs), saponin, antioxidant activity, anti-inflammatory activity

## Abstract

Astaxanthin (AST) exhibits potent antioxidant and anti-inflammatory activities but poor stability and biological efficacy, which limit its application in the food and medical industries. In the present study, a new strategy was proposed to enhance the biological activities of AST using fetal bovine serum-derived extracellular vesicles (EVs). Saponin-assisted incubation was used to load AST owing to its high encapsulation efficiency and loading capacity. AST-incorporated EVs (EV-ASTs) maintained their original EV morphology and showed high stability at 4 °C, 25 °C, and 37 °C over a 28-day period, which was attributed to the protective environment provided by the phospholipid bilayer membrane of the EVs. Additionally, the EV-ASTs exhibited excellent antioxidant and anti-inflammatory activities in HaCaT keratinocytes and RAW 264.7 macrophage cells, respectively; these were significantly higher than those of free AST. Furthermore, the mechanism associated with the enhanced biological activities of EV-ASTs was evaluated by analyzing the expression of genes involved in antioxidation and anti-inflammation, in parallel with cellular in vitro assays. These results provide insights into methods for improving the performance of hydrophobic drugs using nature-derived EVs and will contribute to the development of novel drug-delivery systems.

## 1. Introduction

Astaxanthin (AST) is a biologically active, lipid-soluble pigment mainly found in algae, yeast, and shrimp and is widely used as a nutritional supplement or essential ingredient in the food and medical industries [1,2,3,4,5,6,7]. AST possesses various biological activities, including neuroprotective, anticancer, antidiabetic, antiaging, antioxidant, anti-inflammatory activities, and protective effects against ultraviolet light [8,9,10,11,12]. Notably, the antioxidant and anti-inflammatory activities of AST are regarded as its main biological functions. Recent studies have shown that the antioxidant properties of AST exceed those of representative antioxidants such as vitamin C, suggesting that it exerts a protective effect against both oxidation- and immune-related diseases [3]. However, AST has a highly unsaturated structure with 11 double-conjugated bonds, resulting in instability upon exposure to changes in environmental factors such as temperature, light, air, and pH conditions [13,14]. Additionally, AST, which is mostly present in an esterified form with various fatty acids, is not soluble in aqueous solutions but is readily soluble in organic solvents, such as ethanol, acetone, dimethyl sulfoxide (DMSO), and dimethylformamide [4,15]. Therefore, these issues limit the application of AST in the food and medical industries despite its extraordinary antioxidant activities.

Extracellular vesicles (EVs), with diameter of 50–200 nm, have attracted increasing attention as nanoscale lipid bilayer membrane vesicles owing to their secretion from the endolysosomal pathway in all cell types and enhanced stability in various body fluids, including blood, urine, sweat, and saliva [16,17,18,19,20,21,22,23]. In recent years, numerous efforts have been devoted to using EVs as drug delivery vehicles owing to their various advantageous features, such as good biocompatibility, low immunogenicity and cytotoxicity, increased cellular absorption, specific targeting capability, and ability to cross the blood–brain barrier [24,25,26,27]. In general, the hydrophobic lipid bilayer membrane structure of EVs containing a hydrophilic cavity enables encapsulation of both hydrophilic and hydrophobic drugs into the core and lipid bilayer [28]. This can be performed using different loading methods such as simple incubation, permeabilization, extrusion, freeze-thaw, sonication, and electroporation [29,30]. Importantly, all these methods need to be evaluated to determine the optimal choice with the highest encapsulation efficiency for a specific drug. Furthermore, mammalian cells, which are generally used to produce EVs, are not suitable for this purpose [31]. Moreover, the procedure for obtaining a large number of EVs from mammalian cells is inefficient and time consuming. Instead, fetal bovine serum (FBS), an important and commonly used medium supplement in cell culture, contains many EVs that support cell growth by reducing its sensitivity to both genetic toxicity and endoplasmic reticulum stress [32,33]. Notably, FBS-derived EVs (FBS-EVs) are easily obtained in a cost-effective manner, suggesting that FBS is an excellent source for the production of EVs applicable in pharmaceuticals as a natural drug carrier [34,35,36].

**Scheme 1:**
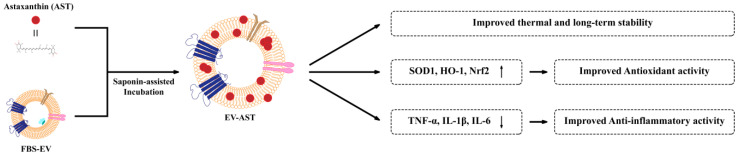
Schematic illustration of the preparation of EV-ASTs and expression levels of genes associated with improved biological activities. EV-AST: Astaxanthin-incorporated extracellular vesicle; SOD1: Superoxide dismutase 1; FBS-EV: Fetal bovine serum-derived extracellular vesicle; HO-1: Heme oxygenase-1; Nrf2: Nuclear factor erythroid 2-related factor 2; TNF-α: Tumor necrosis factor-α; IL-1β: Interleukin-1β; IL- 6: Interleukin-6.

In this study, a novel strategy was devised to improve the biological stability and activity of AST using EVs as drug delivery vehicles (Scheme 1). Specifically, FBS-EVs obtained via size-exclusion chromatography (SEC) were used to incorporate AST, and saponin-assisted incubation was confirmed as the most effective method for the preparation of AST-incorporated EVs (EV-ASTs). The optimized EV-ASTs maintained the EV structure without damage and improved stability at various temperatures, suggesting their capacity for long-term storage. Importantly, the enhanced antioxidant and anti-inflammatory activities of EV-ASTs in cells were validated and the associated biological mechanisms were further investigated by evaluating the expression levels of the genes involved in these activities.

## 2. Materials and Methods

### 2.1. Materials

AST extract from *Blakeslea trispora* was purchased from Sigma-Aldrich (St. Louis, MO, USA). Saponin was purchased from JUNSEI (Chuo-ku, Tokyo, Japan). Cell counting kit-8 (CCK-8) was purchased from Dojindo (Rockville, MD, USA). 2,2-Azinobis (3-ethylbenzothiazoline-6-sulfonic acid) (ABTS), 2′,7′-dichlorofluorescin diacetate (DCFH-DA), 2,2′-azobis (2-methylpropionamidine) dihydrochloride (ABAP), and lipopolysaccharide (LPS) from *Escherichia coli* O111:B4 were purchased from Sigma-Aldrich (St. Louis, MO, USA). Zeba™ spin 7K was purchased from Thermo Fisher Scientific (Waltham, MA, USA). Dimethyl sulfoxide (DMSO) was purchased from Samchun Chemicals (Seoul, Republic of Korea). FBS was purchased from Gibco (Grand Island, NY, USA). Dulbecco’s modified Eagle’s medium (DMEM), Dulbecco’s phosphate-buffered saline (DPBS), and 100× penicillin/streptomycin were purchased from Welgene (Gyeongsan, Republic of Korea).

### 2.2. Cell Culture

Human keratinocytes (HaCaT) from ATCC (Manassas, VA, USA) and mouse macrophages (RAW 264.7) from the Korean Cell Line Bank (Seoul, Republic of Korea) were cultured in DMEM (Welgene, Gyeongsan, Republic of Korea) supplemented with 10% (*v/v*) FBS and 1% penicillin/streptomycin (Welgene, Gyeongsan, Republic of Korea). The cells were maintained at 37 °C with 5% CO_2_ in a humidified incubator (Thermo Fisher Scientific, Waltham, MA, USA).

### 2.3. EV Isolation

20 mL of FBS was used to obtain EVs. Briefly, FBS was subjected to a series of centrifugations at 300× *g*, 2000× *g*, and 10,000× *g* for 5, 20, and 30 min, respectively. The supernatants were then filtered using filters with pore sizes of 0.45 μm and 0.22 μm (Sartorius, Göttingen, Germany) to remove vesicles sized > 200 nm. A Minimate tangential flow filtration (TFF) system with a 300K membrane filter capsule (Pall Corporation, Port Washington, NY, USA) was subsequently used to reduce the volume down to 10 mL. An automatic fraction collector with a qEV10/35-nm column (Izon Science, Christchurch, New Zealand) was used to isolate EVs based on SEC. All isolated EVs (20 mL) underwent another round of centrifugation in a Macrosep 3K membrane (Pall Corporation, Port Washington, NY, USA) at 5000× *g* for 30 min for further concentration down to approximately 10 mL, followed by storage at −80 °C until further use. All centrifugation steps were performed at 4 °C.

### 2.4. Nanoparticle Tracking Analysis (NTA)

The concentration and size distribution of FBS-EVs were analyzed using a NanoSight NS300 (Malvern Panalytical, Malvern, UK). The laser was set at 532 nm, and three 60-s videos were recorded for each sample at 25 frames/s using an sCMOS camera (Thorlabs, Netwon, NJ, USA). For all video recordings, the camera level was set to 14, with a well-adjusted camera focus, and the detection threshold was set to 5. The Brownian movement of EVs was assessed using the NanoSight software (v3.4; Malvern Panalytical, Malvern, UK). For optimal measurements, NTA settings were maintained consistently between samples diluted with 1× phosphate-buffered saline (PBS).

### 2.5. Western Blotting

FBS-EVs, saponin-treated EVs (saponin-EVs), and EV-ASTs were subjected to 10-fold enrichment using a 30K Amicon Ultra-0.5 centrifugal filter (Merck Millipore, Billerica, MA, USA). The EVs were first denatured at 95 °C for 10 min in 1× sodium dodecyl sulfate-polyacrylamide gel electrophoresis loading buffer containing β-mercaptoethanol (Biosesang, Seongnam, Republic of Korea) and resolved on 10% TGX stain-free protein gels using a Mini-Protean tetra system (Bio-Rad, Hercules, CA, USA) at 300 V for 20 min. The resolved proteins were transferred to 0.2-μm polyvinylidene fluoride membranes on a Trans-blot turbo system (Bio-Rad, Hercules, CA, USA) according to the manufacturer’s instructions. Membranes were blocked with 5% bovine serum albumin in Tris-buffered saline with Tween 20 (TBS-T; 1× TBS with 0.1% Tween-20) for 1 h. Primary antibodies (anti-CD63, anti-CD9, anti-Hsp70, anti-Hsp90α, and anti-calnexin; all 1:1000 dilution; ABclonal Technology, Woburn, MA, USA) were incubated with Can Get Signal Solution 1 (Toyobo, Osaka, Japan) for 1 h at room temperature (25 °C). Membranes were then incubated with secondary antibodies (horseradish peroxidase-conjugated goat anti-rabbit IgG; 1:10,000 dilution; ABclonal, Woburn, MA, USA) in Can Get Signal solution 2 (Toyobo, Osaka, Japan) for 40 min at room temperature (25 °C). The bands on the membrane were detected using Clarity Western ECL substrate (Bio-Rad, Hercules, CA, USA) on a ChemiDoc Imaging System (Bio-Rad, Hercules, CA, USA). Washing steps were performed after incubation with primary and secondary antibodies three and four times, respectively, with TBS-T.

### 2.6. Cryo-Transmission Electron Microscopy (Cryo-TEM)

FBS-EVs, saponin-EVs, and EV-ASTs were characterized using cryo-transmission electron microscopy (cryo-TEM). First, a holey carbon-coated copper grid with 200 mesh (Quantifoil, Großlöbichau, Germany) was glow-discharged to make it hydrophilic, and 3 µL of each sample was dropped and vitrified using a Vitrobot (Thermo Fisher Scientific, Waltham, MA, USA) by plunging into liquid ethane. The samples were then stored in liquid nitrogen and transferred to a cryoholder, which was maintained at −180 °C. A JEM-2100 PLUS electron microscope (JEOL, Tokyo, Japan) coupled to a CMOS camera (Thorlabs, Netwon, NJ, USA) at 25 kV was used to obtain TEM images.

### 2.7. Drug-Loading Methods

Simple incubation, saponin-assisted incubation, extrusion, freeze-thaw, sonication, and electroporation were employed to load AST into FBS-EVs. The concentrations of FBS-EVs and AST used in all the loading experiments were fixed at 5 × 10^9^ particles/mL and 200 μg/mL, respectively. For simple incubation, the FBS-EVs were incubated with AST for 2 h at 37 °C. For saponin-assisted incubation, FBS-EVs were incubated with AST in the presence of saponin (0.2%) for 30 min at 37 °C. For extrusion, the mixture of FBS-EVs and AST was extruded twice using an Avanti lipid extruder (Avanti Polar Lipids Inc., Alabaster, AL, USA) with a 200-nm pore filter. For the freeze-thaw method, a mixture of FBS-EVs and AST was rapidly frozen in liquid nitrogen for 15 min and thawed at 37 °C for 15 min, which was repeated three times. For sonication, the mixture of FBS-EVs and AST was sonicated in a water-bath sonicator (SAE HAN ULTRASONIC SH-2140D; 3.3-L capacity, dimensions: 240 × 140 × 100 mm, Seoul, Republic of Korea) at 40 KHz for 30 min on ice, after which the sonicated mixture was incubated at 37 °C for 1 h. For electroporation, a mixture of FBS-EVs and AST was electroporated with two poring pulses (40 V, 1 ms), followed by five transfer pulses (20 V, 50 ms) in a 2-mm gap electroporation cuvette using a Super Electroporator NEPA21 type II (NEPA Genes, Tokyo, Japan). All EV-ASTs prepared using each method were purified using a Zeba spin desalting column (7K; Thermo Fisher Scientific, Waltham, MA, USA) to remove excess AST.

### 2.8. Determination of AST Content in EV-AST

After filtration of EV-ASTs through a Nanosep 300K Omega (Pall Corporation, Port Washington, NY, USA), the EV-ASTs remaining on the membrane were dissolved in DMSO to collect the AST. AST dissolved in DMSO was then filtered and the absorbance was measured at 488 nm (the maximum absorbance peak of AST) using a spectrophotometer (Spectramax iD5 multimode microplate reader; Molecular Devices, Sunnyvale, CA, USA). The encapsulation efficiency (EE) and loading capacity (LC) were estimated using the following equations:

$$\mathrm{EE}\;(\%)=\frac{Loaded\;amounts\;of\;AST\;\left(g\right)}{Initial\;amounts\;of\;AST\;\left(g\right)}\times 100\;\left(\%\right)\;\mathrm{LC}\;(\mathrm{fg}/\mathrm{particle})=\;\frac{Loaded\;amounts\;of\;AST\;\left(\mathrm{fg}\right)}{Number\;of\;exosomes\;\left(\mathrm{particle}\right)}$$

### 2.9. Thermal Stability of EV-ASTs

To evaluate the thermal stability of EV-ASTs, 50 μg/mL of free AST and EV-ASTs were stored at 4 °C, 25 °C, and 37 °C for 28 days. The concentration of each sample was estimated by measuring the absorbance at 488 nm every 7 days and used to calculate the relative stability (%), which was defined as the measured concentration divided by the initial concentration of AST (at day 0) multiplied by 100 (%). 

### 2.10. ABTS Radical-Scavenging Assay

The ABTS assay was used to measure electron transfer to determine the radical-scavenging activity of AST. Briefly, ABTS solution was prepared by mixing 7 mM ABTS and 2.45 mM potassium persulfate in distilled water, followed by incubation in the dark for 12–16 h at room temperature. The absorbance of the ABTS solution at 734 nm was adjusted to 0.7 ± 0.02 by dilution with 1× PBS. Free AST and EV-ASTs at different concentrations (0, 1.5, 2.5, 3.5, and 4.5 μg/mL) were added to the ABTS solution in each well of a 96-well plate and incubated in the dark at room temperature for 6 h. Finally, the absorbance of each mixture was measured at 734 nm using a spectrophotometer (Spectramax iD5; Molecular Devices, Sunnyvale, CA, USA) and the ABTS scavenging activity (%) of AST was calculated using the following equation:

$$\mathrm{Scavenging}\;\mathrm{activity}\;(\%)=\left[1-\frac{As}{Ac}\right]\times 100\;\left(\%\right)$$

where *As* is the absorbance in the presence of each sample (free AST or EV-ASTs) and *Ac* is the absorbance in the absence of the sample.

### 2.11. Cell-Viability Assay

The CCK-8 assay was used to evaluate the cytotoxicity of free AST, EV-ASTs, and EVs. Briefly, HaCaT and RAW 264.7 cells were seeded in 96-well culture plates at densities of 6 × 10^4^ cells/well and 3 × 10^4^ cells/well, respectively, and cultured in DMEM supplemented with FBS and penicillin/streptomycin at 37 °C and 5% CO_2_ for 24 h. Free AST, EV-ASTs, and EVs were added to each well and incubated at 37 °C and 5% CO_2_ for 24 h. The concentrations of free AST, EV-ASTs (0, 0.08, 0.16, 0.4, 0.8, 1.6, and 4 μg/mL), and EVs (0, 0.08, 0.16, 0.4, 0.8, 1.6, and 4 × 10^8^ particles/mL) varied accordingly. Finally, the CCK-8 reagent was added to each well and incubated at 37 °C and 5% CO_2_ for 2 h, followed by measurement of the absorbance of each sample at 450 nm using a spectrophotometer (Spectramax iD5; Molecular Devices, Sunnyvale, CA, USA).

### 2.12. Cellular Antioxidant Activity (CAA) Assay

The CAA assay was performed using DCFH-DA, a cell-permeant indicator for reactive oxygen species (ROS) that is non-fluorescent until its acetate groups are removed by intracellular esterase and oxidized to DCF in the presence of ROS. HaCaT cells were seeded at a density of 6 × 10^4^ cells/well in a 96-well black culture plate and cultured in DMEM supplemented with FBS and penicillin/streptomycin at 37 °C and 5% CO_2_ for 24 h. After removing the cell culture media, each well was treated with 10 μM DCFH-DA diluted in DMEM containing penicillin/streptomycin only, followed by the addition of free AST and EV-ASTs at different concentrations (0.4, 0.6, and 0.8 μg/mL). After incubation for 1 h at 37 °C, the cell culture media was removed and the cells were washed with DPBS. Each well was treated with 600 μM ABAP to induce oxidative stress and incubated for 30 min at 37 °C. Finally, fluorescence intensities were measured every 5 min at excitation and emission wavelengths of 485 and 530 nm, respectively, using a spectrophotometer (Spectramax iD5; Molecular Devices, Sunnyvale, CA, USA). The ROS-scavenging activity of AST was expressed as CAA (%), using the following equation:

$$\mathrm{CAA}\;(\%)=\left[1-\frac{Fc}{Fs}\right]\times 100\;\left(\%\right)$$

where *Fs* is the fluorescence intensity in the presence of each sample (free AST or EV-ASTs) and *Fc* is the fluorescence intensity in the absence of the sample.

### 2.13. Anti-Inflammatory Assay

To measure the anti-inflammatory activity of free AST and EV-ASTs, nitric oxide (NO) concentration produced in RAW 264.7 cells was measured via the Griess reaction. Briefly, RAW 264.7 cells were seeded at a density of 3 × 10^4^ cells/well in 96-well transparent culture plates and cultured in DMEM supplemented with FBS and penicillin/streptomycin at 37 °C and 5% CO_2_ for 24 h. After removal of cell culture media and washing with DPBS, free AST and EV-ASTs at different concentrations (0.4, 0.6, and 0.8 μg/mL) were added to each well and incubated at 37 °C and 5% CO_2_ for 24 h. LPS at a final concentration of 100 ng/mL was added to each well and incubated at 37 °C and 5% CO_2_ for 24 h. The Griess reaction using the Griess reagent system (Promega, Madison, WI, USA) was performed using the supernatant from each well, according to the manufacturer’s instructions, and the absorbance of the Griess reagent mixture was measured at 540 nm using a spectrophotometer (Spectramax iD5; Molecular Devices, Sunnyvale, CA, USA). The NO production (%) was calculated using the following equation:

$$\mathrm{NO}\;\mathrm{production}\;(\%)=\frac{As}{Ac}\times 100\;\left(\%\right)$$

where *As* is the absorbance in the presence of each sample (free AST or EV-ASTs) and *Ac* is the absorbance in the absence of the sample. For both *As* and *Ac*, LPS was used to induce the inflammatory response in RAW 264.7 cells.

### 2.14. mRNA Analysis

After the treatment of HaCaT and RAW 264.7 cells with free AST and EV-ASTs, respectively, at 0.8 μg/mL as described above, mRNA analysis was performed by reverse transcription-quantitative polymerase chain reaction (RT-qPCR). Briefly, total RNA was extracted using a NucleoSpin RNA Plus kit (Macherey-Nagel, Düren, Germany) and reverse-transcribed to synthesize cDNA using a TOPscript cDNA synthesis kit (Enzynomics, Daejeon, Republic of Korea) with 500 ng of the extracted RNA. Gene expression was analyzed by qPCR using gene-specific primers (500 nM), as listed in Table S1. qPCR was performed using 1× TOPreal SYBR Green qPCR PreMIX (Enzynomics, Daejeon, Republic of Korea) under the following conditions: 95 °C for 10 min, followed by 35 cycles of 95 °C for 10 s, 60 °C for 15 s, and 72 °C for 30 s. Relative expression was calculated using the 2^−ΔΔCt^ method, with glyceraldehyde 3-phosphate dehydrogenase (GAPDH) and ribosomal protein lateral stalk subunit P0 (RplpO) levels used as reference genes in HaCaT and RAW 264.7 cells, respectively.

### 2.15. Statistical Analysis

All statistical analyses were performed using the GraphPad Prism software (v9.0; GraphPad Software, Inc., La Jolla, CA, USA). Data are expressed as the means ± standard deviations. Statistical differences between the two samples were assessed using Student’s *t*-test, with *p* < 0.05 considered significant. All experiments were performed in triplicates.

## 3. Results and Discussion

### 3.1. Characterization of FBS-EVs

**Figure 1:**
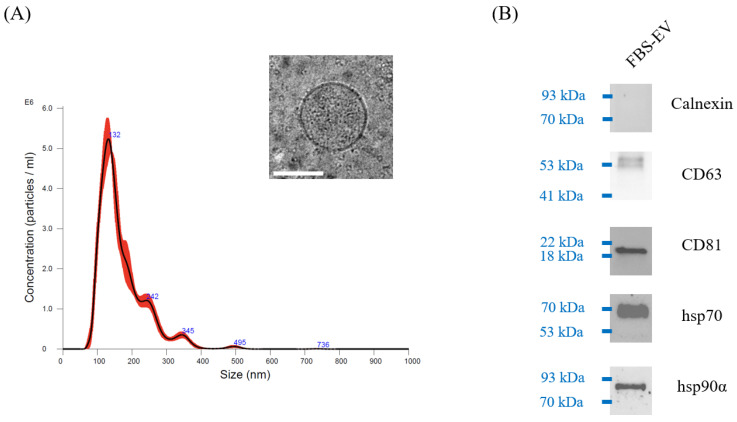
Characterization of FBS-EVs. (**A**) NTA results for FBS-EVs. Cryo-TEM image of FBS-EVs (inset). Scale bar = 100 nm. (**B**) Western blotting results for FBS-EVs.

FBS-EVs were characterized by NTA and cryo-TEM. As shown in the NTA profile (Figure 1A), the average concentration of the FBS-EVs was 5.07 ± 0.05 × 10^10^ particles/mL and the average size of the FBS-EVs was 135 ± 6 nm, which matched the size measured using cryo-TEM (Figure 1A, inset) [37]. Additionally, FBS-EVs exhibited a spherical morphology, displaying a dark internal density with a clearly discernable lipid bilayer. Western blot analyses (Figure 1B) revealed that the FBS-EVs were positive for the pan-EV markers, hsp70, hsp90α, CD63, and CD81 and negative for calnexin, a well-known marker of the endoplasmic reticulum membrane [38,39,40,41]. These findings clearly confirm that sufficient amounts of EVs can be obtained from FBS, which is suitable as a vehicle for AST delivery [34,35,36].

### 3.2. Selection of the Methods Used to Load AST into FBS-EVs

**Figure 2:**
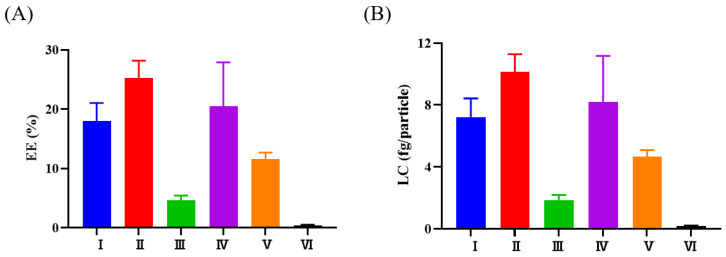
AST-loading methods for EVs. Measurement of (**A**) EE and (**B**) LC for AST loading into EVs using different loading methods. I, simple incubation; II, saponin-assisted incubation; III, extrusion; IV, freeze-thaw; V, sonication; VI, electroporation.

Different loading methods were tested for their ability to incorporate AST into FBS-EVs, including simple incubation, saponin-assisted incubation, extrusion, freeze-thaw, sonication, and electroporation, and determined the optimal method based on the EE and LC results. AST concentration was calculated from the calibration curve obtained using known concentrations of AST (Figure S1). Figure 2A,B show that the highest EE and LC were achieved using saponin-assisted incubation, whereas electroporation resulted in the lowest measurements, which conflicted with findings from a previous report [42,43]. These results emphasize the importance of identifying an appropriate loading method according to the nature of the drugs and delivery vehicles. For example, hydrophilic drugs, such as doxorubicin and paclitaxel, need to pass through the hydrophobic lipid bilayers of EVs; thus, extrusion, sonication, and electroporation could be more efficient for loading such drugs into EVs [30,43,44]. In contrast, the hydrophobicity of AST used in the present study enables its incorporation into the phospholipid bilayer of the EV membrane rather than into the hydrophilic lumen [45,46]. Therefore, it is speculated that extrusion, sonication, and electroporation would overly disrupt the lipid bilayer of EVs, thereby increasing the chance of exposing AST to a hydrophilic environment, consequently leading to inefficient incorporation into the EV membrane. As a mild detergent that induces permeabilization while preserving the integrity of the EV membrane, saponin promotes effective incorporation of AST into the lipid bilayers of EVs [47]. The results confirmed saponin-assisted incubation as the most suitable method for loading AST into EVs, which was used for subsequent experiments.

### 3.3. Characterization of EV-ASTs

**Figure 3:**
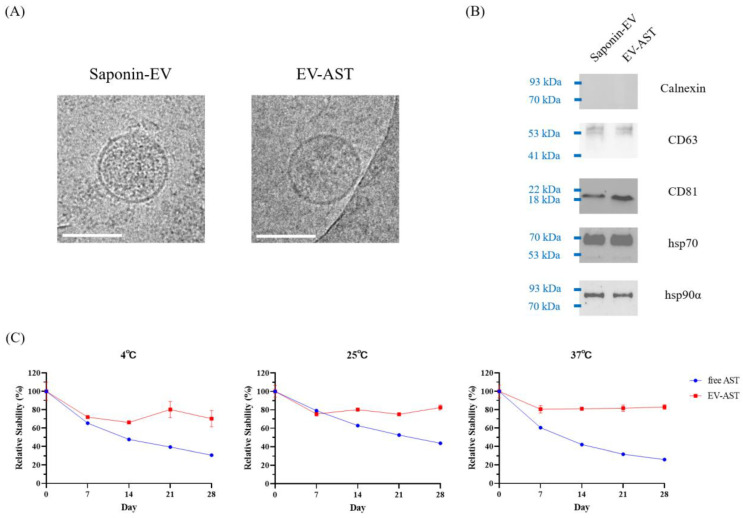
Enhanced stability of AST by loading it into EV. (**A**) Cryo-TEM images of saponin-EVs and EV-ASTs prepared via saponin-assisted incubation. Scale bar = 100 nm. (**B**) Western blotting results for saponin-EVs and EV-ASTs prepared by saponin-assisted incubation. (**C**) Thermal stability of free AST and EV-ASTs at 4, 25, and 37 °C. Relative stability was measured every 7 days for 28 days.

In addition to high loading efficiency, intact EVs with spherical morphology and without membrane disruptions are preferred because these properties are closely associated with improving the thermal stability and biological activities of drug molecules [30,43]. Therefore, saponin-EVs and EV-ASTs prepared via saponin-assisted incubation were characterized using cryo-TEM. Figure 3A shows that the structure of the EV-ASTs was the same as that of intact EVs before and after saponin treatment (Figure 1A, inset). Figure 3B shows that the pan-EV markers, hsp70, hsp90α, CD63, and CD81 were positive for both saponin-EVs and EV-ASTs prepared by saponin-assisted incubation. Similarly, calnexin was negative for both EVs. These results confirmed that both saponin and AST exerted no effect on the size, morphology, and characteristics of the EVs, suggesting that saponin-assisted incubation is a reliable loading method for AST.

Additionally, the protective effect of EVs on AST stability was evaluated by monitoring the relative stability of free AST after storage at different temperatures (4 °C, 25 °C, and 37 °C) and periods (0, 7, 14, 21, and 28 days). Figure 3C shows that the EV-ASTs retained up to 80% of AST stability at 7 days, with this level remaining consistent for 28 days, whereas free AST stability was degraded over time, with <40% of the original stability remaining at 28 days. These results demonstrate the enhanced stability of EV-ASTs in terms of their thermal capacity and long-term storage. It was speculated that EVs provided a protective environment for AST, which is consistent with the increase in the stability of other biological molecules, including nucleic acids and proteins, by EVs [36,48,49].

### 3.4. In Vitro Antioxidant Activity of Free AST and EV-ASTs

**Figure 4:**
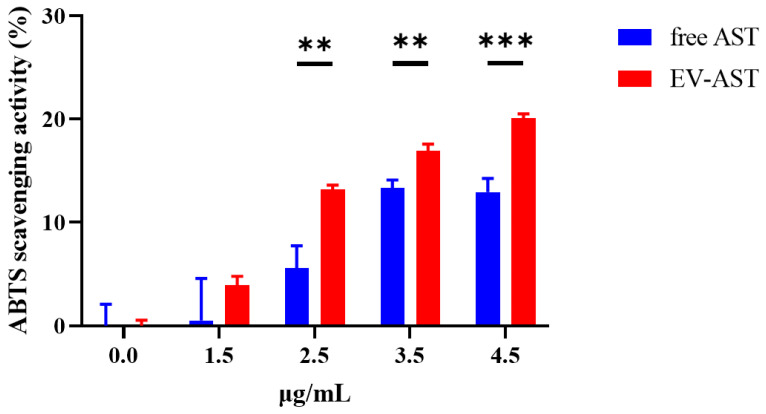
ABTS radical scavenging activity (%) of free AST and EV-AST at concentrations of 0, 0.5, 1.5, 2.5, 3.5, 4.5, and 5 μg/mL (**: *p* < 0.01; ***: *p* < 0.001).

The in vitro antioxidant activity of EV-ASTs was investigated and compared with that of free AST via the ABTS radical scavenging assay. In principle, antioxidants suppress the formation of colored ABTS radicals, an oxidized form of ABTS produced by powerful oxidants such as potassium persulfate, by reducing ABTS radicals to non-colored ABTS [50,51]. As shown in Figure 4, various concentrations of free AST and EV-ASTs demonstrated concentration-dependent antioxidant activity, with EV-ASTs exerting 136%, 27%, and 55% higher levels of antioxidant activity than free AST at 2.5, 3.5, and 4.5 μg/mL (*p* < 0.01, 0.01, and 0.001, respectively). It was speculated that the enhanced antioxidant activity of EV-ASTs was a consequence of the stabilizing effect of EVs on AST (Figure 3B).

### 3.5. Evaluation of Cytotoxicity in HaCaT and RAW 264.7 Cells

**Figure 5:**
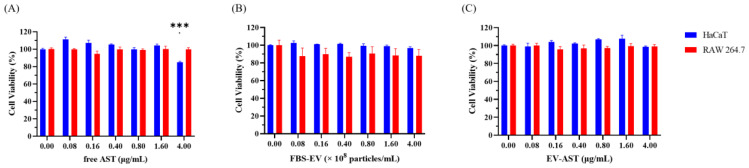
Evaluation of cytotoxicity in HaCaT and RAW 264.7 cells. The viability of HaCaT and Raw 264.7 cells in the presence of (**A**) free AST (0, 0.08, 0.16, 0.4, 0.8, 1.6, and 4 μg/mL), (**B**) FBS-EVs (0, 0.08, 0.16, 0.4, 0.8, 1.6, and 4 × 10^8^ particles/mL), and (**C**) EV-ASTs (0, 0.08, 0.16, 0.4, 0.8, 1.6, and 4 μg/mL) (***: *p* < 0.001).

The cytotoxicity of free AST, FBS-EVs, and EV-ASTs on HaCaT and RAW 264.7 cells was measured via the CCK-8 assay. Figure 5A shows that RAW 264.7 cell viability was unaffected by free AST, whereas that of HaCaT cells was adversely affected by concentrations ≥ 4.0 μg/mL (*p* < 0.001). Additionally, Figure 5B shows that FBS-EVs had no cytotoxic effect on HaCaT or RAW 264.7 cells at all concentrations, which may be attributed to the excellent biocompatibility of FBS-EVs [26,27]. It should be noted that the particle concentrations of FBS-EVs were equal to those of EV-ASTs. Significant cytotoxicity was not observed in HaCaT or RAW 264.7 cells at all EV-AST concentrations (Figure 5C). It was speculated that the biocompatibility of EV protected the cells from the cytotoxicity of free AST.

### 3.6. The CAA of Free AST and EV-ASTs

**Figure 6:**
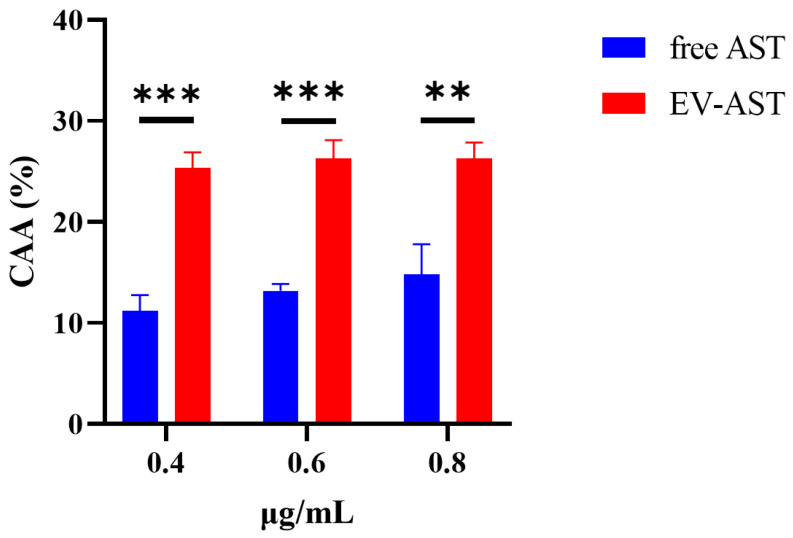
Measurement of CAA in free AST and EV ASTs. The CAA (%) of free AST and EV-AST was determined at different concentrations (0.4, 0.6, and 0.8 μg/mL) in HaCaT cells (**: *p* < 0.01; ***: *p* < 0.001).

CAA assays were performed to measure the antioxidant activity in HaCaT cells. In this assay, cell-permeable DCFH-DA, an oxidation-sensitive indicator, is converted to DCFH by cellular esterase and then oxidized to fluorescent DCF by ROS induced in the presence of ABAP [52]. However, this process is prevented by the presence of antioxidants, which results in a decreased fluorescence signal. In this study, the CAA (%) of free AST and EV-ASTs was estimated at various concentrations (0.4, 0.6, and 0.8 μg/mL). Figure 6 shows that the CAA (%) of EV-ASTs was significantly higher than that of free AST at all concentrations (*p* < 0.01). FBS-EVs showed no significant effects on CAA at any concentration (Figure S2), indicating that CAA were exclusively induced by EV-incorporated AST. It was expected that the higher in vitro antioxidant activity of EV-ASTs relative to that of free AST was a consequence of the efficient delivery of AST into the cells after encapsulation within the EVs, resulting in enhanced cellular antioxidant activity.

### 3.7. The Anti-Inflammatory Activity of Free AST and EV-AST

**Figure 7:**
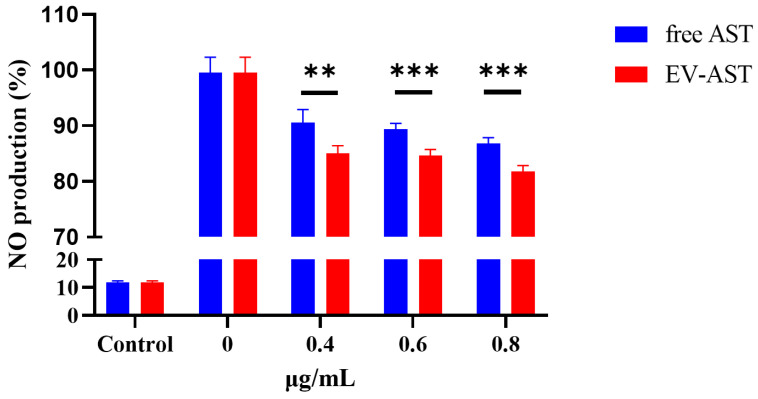
Measurement of the anti-inflammatory activities of free AST and EV ASTs. The anti-inflammatory activities of free AST and EV-ASTs (0.4, 0.6, and 0.8 μg/mL) were determined in LPS-stimulated RAW 264.7 cells (**: *p* < 0.01; ***: *p* < 0.001).

Antioxidants have been reported to exert anti-inflammatory activity owing to the close relationship between these activities [53]. As a strong antioxidant, AST also exhibits anti-inflammatory activity [9,12]. Thus, the anti-inflammatory activities of free AST and EV-ASTs in RAW 264.7 cells were evaluated after the exposure of the cells to LPS to induce an inflammatory response, which was denoted by the increased production of NO. Figure 7 shows that EV-ASTs demonstrated higher anti-inflammatory activity than free AST at all concentrations (*p* < 0.01), which was consistent with the results of the CAA assay (Figure 6). FBS-EVs also showed no significant effects on anti-inflammatory activity at all concentrations (Figure S3). It can be reasoned that the enhancements in biological activities achieved by loading AST into FBS-EVs are not due to the activities of FBS-EVs themselves, but solely due to the enhanced delivery of AST in FBS-EVs into cells through the endocytic pathway [54,55]. 

### 3.8. Assessment of Gene Expression Related to the Antioxidant and Anti-Inflammatory Activities of the EV-ASTs

**Figure 8:**
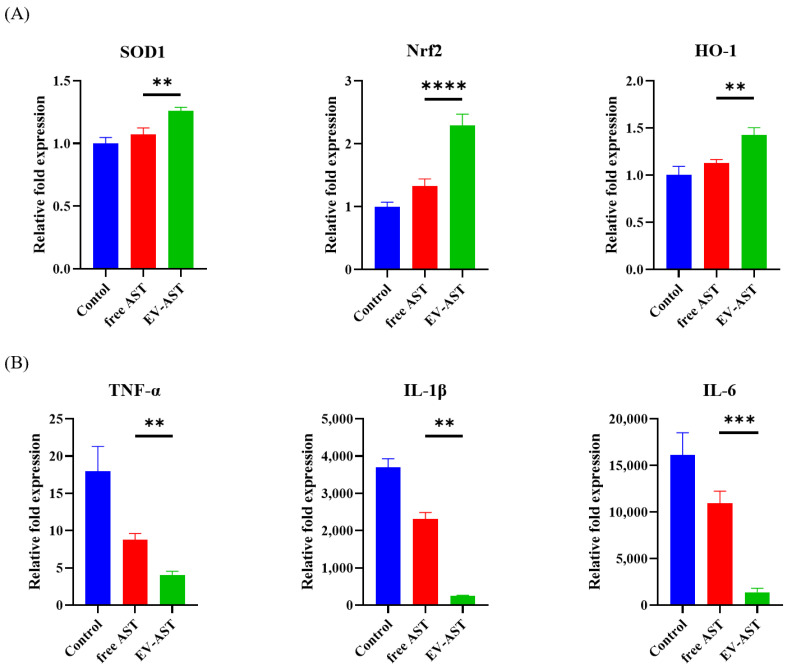
Determination of the altered expression of genes related to antioxidant and anti-inflammatory activities. Gene expression related to (**A**) antioxidant and (**B**) anti-inflammatory activities of free AST and EV-ASTs (0.8 μg/mL) in HaCaT and LPS-stimulated RAW 264.7 cells, respectively (**: *p* < 0.01; ***: *p* < 0.001; ****: *p* < 0.0001).

To investigate the biological mechanism associated with the antioxidant and anti-inflammatory activities of EV-ASTs, the expression of genes related to each activity was analyzed using RT-qPCR. To assess changes associated with antioxidant activity in HaCaT cells, the expression of superoxide dismutase 1 (SOD1), nuclear factor erythroid 2-related factor 2 (Nrf2), and heme oxygenase-1 (HO-1), which play important roles in cellular oxidation was evaluated [56,57,58]. As shown in Figure 8A, the expression of SOD1, Nrf2, and HO-1 genes increased in the presence of both free AST and EV-ASTs. However, the expression of SOD1, Nrf2, and HO-1 genes was 18%, 73%, and 27% higher, respectively, in the presence of EV-ASTs than in the presence of free AST, suggesting a higher degree of antioxidant activity (*p* < 0.01, 0.0001, and 0.01, respectively). As the expression of SOD1, Nrf2, and HO-1 genes leads to the inhibition of ROS generation, these results support the higher antioxidant activity of EV-AST than free AST.

To evaluate the effects of AST-specific anti-inflammatory activity, changes in the expression of pro-inflammatory cytokines, tumor necrosis factor (TNF)-α, interleukin (IL)-1β, and IL-6 in RAW 264.7 cells were measured after LPS stimulation [59,60]. As shown in Figure 8B, the expression of all three genes decreased in the presence of free AST and EV-ASTs; this decrease was more substantial in the presence of EV-ASTs (*p* < 0.01). As the expression of these cytokines leads to inflammation, decreased expression shows that EV-ASTs inhibit the intermediate process of the inflammation pathway, supporting the results shown in Figure 7.

## 4. Conclusions

In summary, a novel strategy was demonstrated to improve the thermal stability, long-term storage, and biological activity of AST using FBS-EVs. In addition to the systematic characterization of FBS-EVs, an optimal AST-loading method with preservation of EV characteristics and AST activity was established. EV-ASTs were demonstrated to result in the enhanced antioxidant and anti-inflammatory activities, compared to those of free AST, and mRNA analyses offered insights into the biological mechanisms associated with EV-AST activity; however, there would be possible adverse effects of EV-ASTs such as unexpected cytotoxicity to other cells, allergic reactions, and stomach pain. Furthermore, the mechanism of AST delivery by EV should be further studied. Nonetheless, these findings provide a foundation for enhancing the biological activities of AST using EVs as drug delivery vehicles and promoting the wider application of AST in food supplements and functional ingredients.

## Data Availability

The data are contained within this article.

## Supplementary Materials

The following supporting information can be downloaded at: https://www.mdpi.com/article/10.3390/antiox12020473/s1, Figure S1: Measurement of the concentration of astaxanthin. (A) Absorbance spectrum curve of 50 μg/mL AST. (B) Standard curve of AST (0.5–50 μg/mL); Figure S2: Measurement of the CAA of FBS-EVs (0.4, 0.6, and 0.8 × 10^8^ particles/mL) in HaCaT cells; Figure S3: Measurement of the anti-inflammatory activities of FBS-EVs (0.4, 0.6, and 0.8 × 10^8^ particles/mL) in LPS-stimulated RAW 264.7 cells; Figure S4: Cryo-TEM images at lower magnification of (A) FBS-EV (Figure 1A), (B) saponin-treated FBS-EVs (Figure 3A), and (C) EV-ASTs prepared via saponin-assisted incubation (Figure 3A). Scale bar = 200 nm; Table S1: Primer sequences for RT-qPCR experiments.
