# Supplementary material for: Extracellular Vesicles, as Drug-Delivery Vehicles, Improve the Biological Activities of Astaxanthin
Source: Antioxidants (Basel). 2023 Feb 13;12(2):473. doi: 10.3390/antiox12020473 (PMC9952194; doi:10.3390/antiox12020473)
Supplement: Supplementary file 1 [file antioxidants-12-00473-s001.zip › antioxidants-2208651-supplementary.pdf]

antioxidants

Article

Extracellular vesicles, as drug-delivery vehicles, improve the biological activities of astaxanthin

Young Jun Jang †, Byung Seok Cha †, Doyeon Kim, Eun Sung Lee, Seokjoon Kim, Jinjoo Han, Jiye Shin, Seokhwan Kim and Ki Soo Park *

Department of Biological Engineering, College of Engineering, Konkuk University, Seoul 05029, Republic of Korea; alan0922@konkuk.ac.kr (Y.J.J.); cbs934@konkuk.ac.kr (B.S.C.); garamag@konkuk.ac.kr (D.K.); afish94@konkuk.ac.kr (E.S.L.); ghjghy@konkuk.ac.kr (S.K.); jinjoo9665@konkuk.ac.kr (J.H.); tlswldp0911@konkuk.ac.kr (J.S.); mmm1605@konkuk.ac.kr (S.K.)

* Correspondence: akdong486@konkuk.ac.kr; Tel.: +82-2-450-3742

† These authors equally contributed to this work.

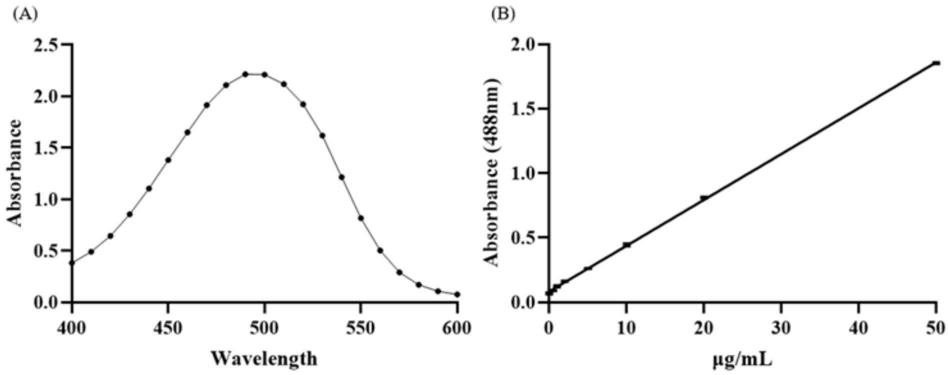

Figure S1. Measurement of the concentration of astaxanthin. (A) Absorbance spectrum curve of 50 μg/mL AST. (B) Standard curve of AST (0.5–50 μg/mL).

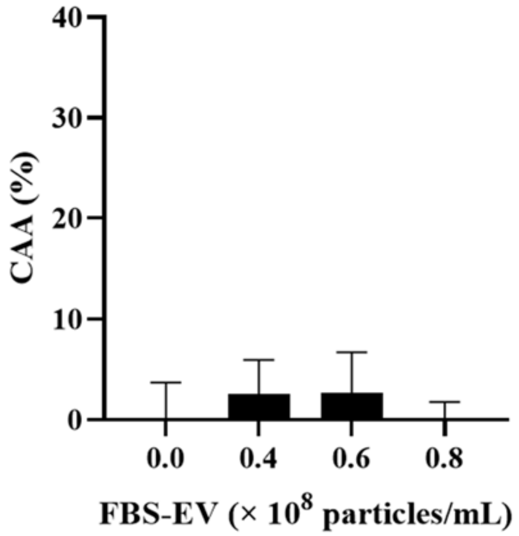

Figure S2. Measurement of the CAA of FBS-EVs (0.4, 0.6, and 0.8 × 10^8 particles/mL) in HaCaT cells.

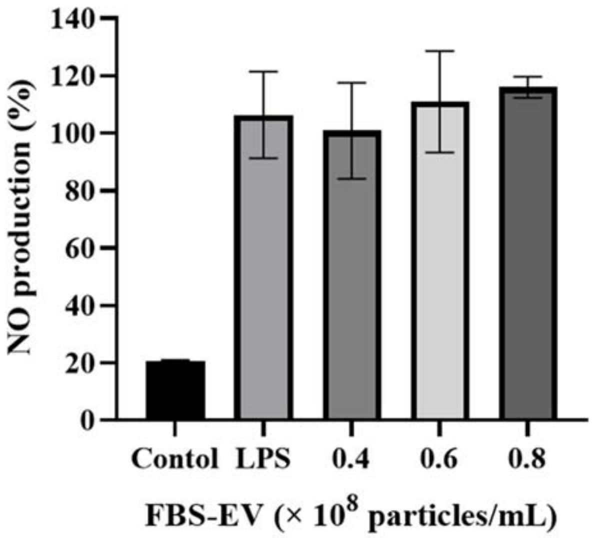

Figure S3. Measurement of the anti-inflammatory activities of FBS-EVs (0.4, 0.6, and 0.8 × 10^8 particles/mL) in LPS-stimulated RAW 264.7 cells.

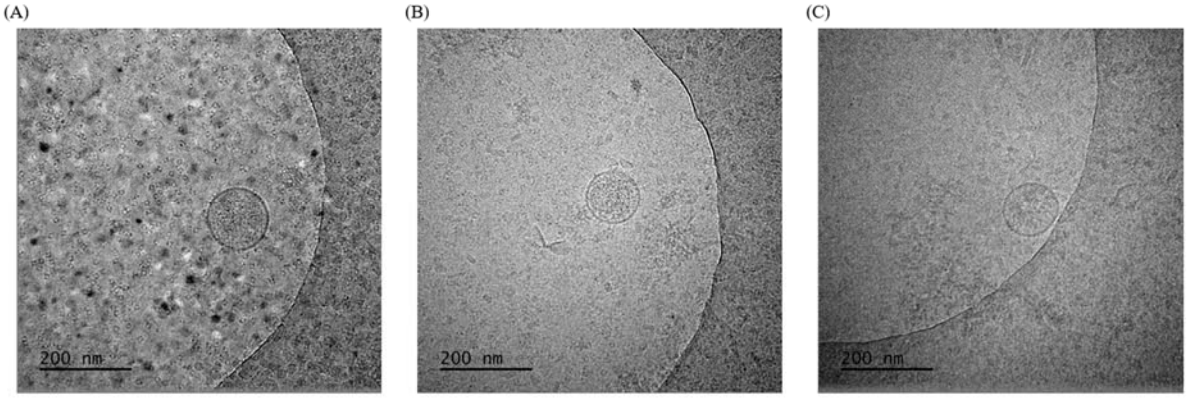

Figure S4. Cryo-TEM images at lower magnification of (A) FBS-EV (**Fig. 1A**), (B) saponin-EVs (**Fig. 3A**), and (C) EV-ASTs prepared via saponin-assisted incubation (**Fig. 3A**). Scale bar = 200 nm.

Table S1. Primer sequences used for the RT-qPCR experiments.

Gene	Forward Primer (5′–3′)	Reverse Primer (5′–3′)
GAPDH	GCCCAGATGGATATGGTGAA	ATGGGACGGTTCACATGTTC
SOD1	AGGCTGTACCAGTGCAGGTC	CAATAGACACATCGGCCACA
Nrf2	ACATCCTTTGGAGGCAAGAC	TCGGGTCATTGTGAGTCAGT
HO-1	CAAGGAGAGCCCAGTCTTCG	CTGGTGTGTAGGGGATGACC
Rplp0	TGAACATGCTGAACATCT	TATAAATGCTGCCGTTGT+
TNF-α	GGCTGCCCCGACTACGT	ACTTTCTCCTGGTATGAGATAGCAAAT
IL-1β	GTCACAAGAAACCATGGCACAT	GCCCATCAGAGGCAAGGA
IL-6	CTGCAAGAGACTTCCATCCAGTT	AGGGAAGGCCGTGGTTGT
